# Retrospective analysis of the impact of respiratory motion in treatment margins for frameless lung SBRT based on respiratory‐correlated CBCT data‐sets

**DOI:** 10.1002/acm2.13034

**Published:** 2020-09-30

**Authors:** Sheeba Thengumpallil, Damien Racine, Jean‐François Germond, Nicolas Péguret, Jean Bourhis, François Bochud, Raphaël Moeckli

**Affiliations:** ^1^ Institute of Radiation Physics Lausanne University Hospital Lausanne Switzerland; ^2^ Clinique des Grangettes Chêne‐Bougeries Switzerland; ^3^ Department of Radiation Oncology Lausanne University Hospital Lausanne Switzerland

**Keywords:** 4D CBCT, 4D CT, breathing motion, bootstrap, margins

## Abstract

**Purpose:**

To investigate the impact of respiratory motion in the treatment margins for lung SBRT frameless treatments and to validate our treatment margins using 4D CBCT data analysis.

**Methods:**

Two hundred and twenty nine fractions with early stage NSCLC were retrospectively analyzed. All patients were treated in frameless and free breathing conditions. The treatment margins were calculated according to van Herk equation in Mid‐Ventilation. For each fraction, three 4D CBCT scans, pre‐ and postcorrection, and posttreatment, were acquired to assess target baseline shift, target localization accuracy and intra‐fraction motion errors. A bootstrap analysis was performed to assess the minimum number of patients required to define treatment margins.

**Results:**

The retrospectively calculated target‐baseline shift, target localization accuracy and intra‐fraction motion errors agreed with the literature. The best tailored margins to our cohort of patients were retrospectively computed and resulted in agreement with already published data. The bootstrap analysis showed that fifteen patients were enough to assess treatment margins.

**Conclusions:**

The treatment margins applied to our patient’s cohort resulted in good agreement with the retrospectively calculated margins based on 4D CBCT data. Moreover, the bootstrap analysis revealed to be a promising method to verify the reliability of the applied treatment margins for safe lung SBRT delivery.

## 1 INTRODUCTION

The application of the Stereotactic Body Radiation Therapy (SBRT) to the lung is a complicated scenario because respiratory‐induced tumor motion has an impact on the target localization and thus influences the accuracy of imaging, treatment planning, and treatment delivery.^1^ One way to reduce these uncertainties is to use a respiratory‐correlated CT scan (4D CT)^2^, ^3^, ^4^, ^5^, ^6^, ^7^ at the treatment preparation stage and to perform 4D cone beam CT (4D‐CBCT) prior to treatment, as both techniques provide 3D images at multiple phases of the respiratory cycle. For lung SBRT in free breathing conditions, a moving organ can be treated using the Mid‐Ventilation approach (Mid‐V),^8^, ^9^ in which the predominant breathing phase closest to the time‐weighted mean tumor position is selected for the planning, thus ensuring small treatment margins. However, when calculating dose and appropriate margins for lung SBRT, motion, baseline shifts and low‐density media must also be considered. Van Herk et al^10^ proposed a probabilistic approach to create the margins. The key of this approach is to include the systematic and random errrors based on clinical practice when computing the margins, in order to ensure adequate tumor dose coverage for a high percentage of patients. The systematic and random errors for lung tumors can be defined by statistical analysis of the distribution of changes in tumor position during a course of radiotherapy. Therefore, the 4D CBCT^11^ makes it possible to assess, prior to each fraction treatment, the mean position and the 3D trajectory of the tumor, and then to verify the applied treatment margins on a daily basis. Moreover, breathing‐related variations in tumor positions can appear during the treatment intra‐ and inter‐fractionally. The intra‐fraction variation describes the variation in the tumor position during one treatment fraction,^12^, ^13^, ^14^ while the inter‐fraction variation describes the variation of the tumor position between fractions. These two types of tumor motion variations are mainly manifested as tumor baseline shifts and introduce additional errors in the treatment. For this reason, 4D CBCT images taken at different moments in the treatment session are an appropriate way to quantify such errors; they also allows to track the 3D trajectory of the tumor, therefore veryfing the applied treatment margins on a daily basis. Within this context, the van Herk approach is commonly used to prospectively define setup margins for lung SBRT. However, this approach relies on statistical analysis of prospective data, assuming that no changes in the process occurs in the future. In this paper we propose two approaches that proactively re‐examines the margins when the process is changed by a modification in contention, treatment technique, etc. In those cases, it is indeed crucial to verify the applied margins to ensure safe SBRT delivery.

## 2 MATERIAL AND METHODS

### 2.1 Patients

Thirty patients (for a total of 229 fractions) with lower (n = 6), upper (n = 9) and mid‐lobe lesions (n = 15) and stage T1‐T2 nonsmall‐cell lung cancer were treated with SBRT. For each patient, a planning CT was acquired (16‐slices Aquilion LB; Toshiba, Japan) using a 4D acquisition protocol. Ten respiratory phases were retrospectively reconstructed from the 4D CT with the phase sorting algorithm. All patients were scanned in head‐first supine position using an arm and knee support. No additional immobilization devices were used, and none of the patients received any instructions for regular breathing before scan and treatment. The tumor motion characteristics, described as amplitude and trajectory, were assessed in the three directions. To determine the Mid‐V phase, 10 GTVs were contoured on the ten phases and the center of the mass of the GTV was determined for each phase. The phase providing the minimum distance between the center of mass of the GTV on each phase and the average center of mass of the GTV among the ten phases was selected as the Mid‐V phase. Once the Mid‐V^9^ phase was identified then it was used for the delineation of the tumor and organs at risk as well as treatment planning. The patient‐specific tumor motion characteristic was used to calculate planning target volume (PTV) margins, with the van Herk equation.^10^

### 2.2 Target volumes and planning

The Gross Target Volume (GTV) was delineated on the Mid‐V phase, in the Velocity software™ version 2.8 (Varian Medical Systems, USA), using a pulmonary level window (WW: 1224; WL: −412). No margins for microscopic extension were added to the GTV to form a Clinical Target Volume (CTV).^15^ The PTV was created by applying margins to the GTV based on the van Herk equation^10^ (see Margins section). The fractionation schemes were 12 x 5 Gy (n = 3), 5 × 12 Gy (n = 6), 8 × 7.5 Gy (n = 20) and 3 × 18 Gy (n = 1) depending on the tumor size and localization. All patients were planned with MONACO Monte Carlo‐based Treatment Planning System (TPS) (Elekta, Sweden) for Volumetric Modulated Arc Therapy (VMAT) delivery. Plans were prepared for a single arc or two arc VMAT deliveries depending on the patient anatomy.

### 2.3 Target motion assessment

Target motion amplitude was individually quantified for all patients before the start of the treatment (Table 1). The amplitude of the target measured from the 4D CT was defined as the maximum coordinates minus the minimum coordinates of the target centroid in the left‐right (LR), cranial‐caudal (CC), and anterior‐posterior (AP) directions. The 3D scalar amplitude was defined as the distance in 3D coordinates and calculated as $\sqrt{{\text{LR}}^{2}+{\text{AP}}^{2}+{\text{SI}}^{2}}$. The 4D CT parameters were 120 kV, 150 mA, a detector width of 320 mm and a rotation time of 0.5 seconds. The pitch was less than 0.1 in order to ensure that at least one complete respiratory cycle was included in the scan acquisition.^16^ Images were reconstructed with 2 mm slice thickness and 2 mm slice separation. The respiratory signal was tracked with the AZ‐733 V ANZAI belt (ANZAI Medical Solutions, Japan) measuring the variation of pressure on the belt generated by the breathing motion. Ten respiratory breathing phases were sorted retrospectively.

**Table 1:** Target motion amplitude at 4D CT.

# Patient	LR [cm]	AP [cm]	SI [cm]	3D [cm]
1	0.2	0.2	0.06	0.29
2	0.21	0.43	0.37	0.6
3	0.09	0.46	1.32	1.4
4	0.04	0.14	0.55	0.57
5	0.12	0.25	1	1.04
6	0.4	0.39	0.2	0.59
7	0.12	0.23	0.1	0.28
8	0.1	0.19	0.27	0.34
9	0.11	0.22	0.2	0.32
10	0.26	0.26	0.12	0.39
11	0.11	0.34	0.8	0.88
12	0.32	0.21	0.2	0.43
13	0.12	0.38	0.2	0.45
14	0.25	0.51	0.4	0.69
15	0.13	0.27	0.2	0.36
16	0.21	0.32	0.67	0.77
17	0.28	0.37	0.3	0.55
18	0.65	0.64	0.4	1
19	0.22	0.23	0.35	0.47
20	0.09	0.22	1.39	1.41
21	0.1	0.2	0.33	0.4
22	0.11	0.33	1.2	1.25
23	0.28	0.26	0.6	0.71
24	0.1	0.2	1.18	1.2
25	0.22	0.21	1.2	1.24
26	0.21	0.11	2.2	2.21
27	0.59	0.19	0.08	0.62
28	0.43	0.21	0.6	0.77
29	0.16	0.17	0.26	0.35
30	0.28	0.25	0.34	0.51

Abbreviations: AP, Anterior‐Posterior target motion amplitude; LR, Left‐Right target motion amplitude; SI, Superior‐Inferior target motion amplitude.

### 2.4 On‐line 4D CBCT for target localization and motion characterization

Image Guided Radiotherapy (IGRT) and treatment were performed on an Elekta Synergy linear accelerator with integrated CBCT scanner (Elekta, Sweden). Each patient was positioned with the arm and knee support used for the planning CT and aligned to the room lasers. A pretreatment 4D CBCT^17^ scan was acquired. The average 4D CBCT was registered to the primary image Mid‐V using XVI software v 4.5 (Elekta, Sweden); an automatic rigid registration of the bony anatomy (i.e. vertebrae) and of the GTV (soft‐tissue) with an expansion of 5 mm was chosen in order to create a 3D shape of the region of interest (ROI). That ROI was automatically registered to each phase of the 4D CBCT. The goal was to verify that the overall tumor motion excursion measured on the ten phases was within the PTV margins applied on the Mid‐V phase. In case a shift was detected, only the translation corrections relative to the GTV isocenter were applied to the couch, because rotation corrections were not supported by our treatment couch. A second 4D CBCT was acquired after couch correction to assess the residual setup errors. Again, a dual automatic registration of both bony anatomy and ROI was performed in order to visually validate the target alignment. A posttreatment 4D CBCT scan was acquired at the end of the treatment delivery to quantify the intra‐fraction motion errors, obtained as the difference in target alignment between the second and the third 4D CBCT scan. To reduce the additional dose to the patient, the first 4D CBCT was acquired with the standard protocol of 4 minutes, while the second 4D CBCT was reduced to 2 minutes by downsizing the number of projections and the final 4D‐CBCT to only 1 minute. The resulting image quality was good enough to evaluate the intra‐fraction motion errors.^18^ Finally, the 3D couch shifts from the three 4D CBCTs using soft‐tissue and bony registration were collected for all treatment fractions and for all patients and used to retrospectively determine the margins according to our data as described in the Margins section.

### 2.5 Margins

The 3D couch shifts using soft‐tissue and bony registration were collected for all treatment fractions for all patients. The mean and standard deviation were calculated for each patient. From these data, the standard deviation of the systematic error (∑), and standard deviation of the random error (σ) were calculated. The ∑ was obtained by calculating the standard deviation of the mean for each patient, and the σ was the root mean square of the standard deviations for each patient. To analyze the difference between the bony and soft‐tissue registration, the couch shifts obtained using soft‐tissue registration were subtracted from the couch shifts obtained using bony registration in each direction. Finally, the PTV margins, M(PTV), for frameless SBRT patients with online 4D CBCT image guidance were calculated for each patient by applying the van Herk equation^10^:

(1) $$M\left(\text{PTV}\right)=2.5\sum \text{TOT}+2.34\sqrt{\sigma_{\text{TOT}}^{2}+\sigma_{p}^{2}}‐2.34\sigma_{p}$$

where ∑_TOT_ and σ_TOT_ are the overall systematic and random errors (as defined in Equations 2 and 3)^19^ and σ_p_ is the width of the penumbra modeled by a cumulative Gaussian.

(2) $$\Sigma_{\text{TOT}}=\sqrt{\Sigma_{\text{delineation}}^{2}+\Sigma_{\text{localisation}}^{2}+\Sigma_{\text{intra}‐\text{fraction}}^{2}}$$

Sum of the standard deviations of the systematic errors calculated from delineation, localization and intra‐fraction.

(3) $$\sigma_{\text{TOT}}=\sqrt{\sigma_{\text{localisation}}^{2}+\sigma_{\text{intra}‐\text{fraction}}^{2}+\sigma_{\text{motion}}^{2}}$$

Sum of the root mean square of the random errors calculated from localization, intra‐fraction and respiratory motion.

For the lung, σ_p_ was taken equal to 0.64 cm as analytically estimated by Mexner et al.^20^ Equation 1 describes the GTV to PTV margin necessary to ensure that the GTV receives 99 % of the prescribed dose for 90 % of the patients when all geometric uncertainties are included. The target delineation error (1 SD = 2 mm for T1‐T2 tumor staging),^21^, ^22^ the target localization error and the intra‐fraction motion error were considered as systematic errors. The latter comes from the fact that the treatment lasts longer than the planning CT and therefore the free breathing of the patient causes a deviation in the shape of the total dose distribution. For each patient the respiratory motion error is calculated according to the Lujan equation.^23^ The margins used for the patients treatment were initially taken from the data of systematic and random errors reported in the literature^17^ (target localization accuracy, intra‐fraction motion and target delineation uncertainty, intra‐fraction motion and respiratory motion, see Table 2). Then the errors obtained from the retrospective analysis of the patients treated at our institution were used to compute customized margins.

**Table 2:** Systematic and random errors of van Herk equation for margins calculation based on the off‐line analysis (4D CBCT data) and from literature (treatment); the latter was taken as reference. A is the target motion amplitude.

	LR (cm)	AP (cm)	SI (cm)
Systematic errors ∑_e_			
Localisation			
Off‐line	0.09	0.15	0.12
Treatment	0.08	0.09	0.08
Delineation			
Off‐line	0.2	0.2	0.2
Treatment	0.2	0.2	0.2
Intra‐fx			
Off‐line	0.03	0.07	0.12
Treatment	0.12	0.18	0.12
Random errors σ_e_			
Localisation			
Off‐line	0.14	0.15	0.2
Treatment	0.11	0.14	0.11
Target motion			
Off‐line	0.36xA(LR)	0.36xA(AP)	0.36xA(SI)
Treatment	0.36xA(LR)	0.36xA(AP)	0.36xA(SI)
Intra‐fx			
Off‐line	0.14	0.15	0.22
Treatment	0.13	0.18	0.15

### 2.6 Iterative analysis

To assess the robustness of our data, the margins were recalculated with an iterative method for different subsets of 10, 15, 20, 25 and 30 patients taken in chronological order (p + 1) with respect to their treatment. The scope was to determine whether or not the margins calculated were converging for samples with increasing number of patients. This approach has been chosen at first due to its rather simple implementation; however it presents a major drawback, the lack of large data to build good statistics. This was a clear limitation to validate our results.

### 2.7 Bootstrap analysis

The principle of the bootstrap method is that inference regarding a population can be modeled by resampling with replacement a subset of this population.^24^, ^25^ In this way, it is possible to expand the sampling from a limited pool of data and better estimate the statistical parameters of the population. In particular, for each patient error (target localisation accuracy, intra‐fraction motion, and target delineation), a resampling was computed with different cohort sizes to create new systematic and random errors. For the bootstrap calculation the delineation systematic error was fixed at 2 mm for all patients. The localization systematic error was defined as: Σ_loc_ = SD (M(p)) where SD represents the standard deviation, M represents the mean, and p represents the p^th^ patient. The p^th^ is a patient created from a resampling with replacement of the localization patient’s data of one patient randomly chosen. The intra‐fraction systematic error was defined as Σ_intra_ = SD (M(s)) where s represents the s^th^ session. The s^th^ is a session created from a resampling with replacement of the intra‐fraction patient’s data of n patients randomly chosen.

The motion amplitude random error was fixed at 0.36 x patient’s motion amplitude (A). The localization random error was defined as $\sigma_{\text{loc}}=\sqrt{\frac{\sum_{p=1}^{n}SD_{p}}{n}}$ where n represents the number of patient and p represents the p^th^ patient. Finally, the intra‐fraction random error was defined as: $\sigma_{\text{intra}}=\sqrt{\frac{\sum_{s=1}^{n}SD_{s}}{n}}$ where n represents the number of patient and s represents the s^th^ session.

In our experiment, the cohort size was defined by the number of patients n included in this cohort with n equal to 3,6,9,12,15,30,50,75,100 and 150. For each sampling, bootstrap calculation was repeated 1000 times with random choice of patients at each time. The mean and standard error was calculated from the results obtained with these 1000 repetitions.

## 3 RESULTS

### 3.1 Target motion

**Fig. 1:**
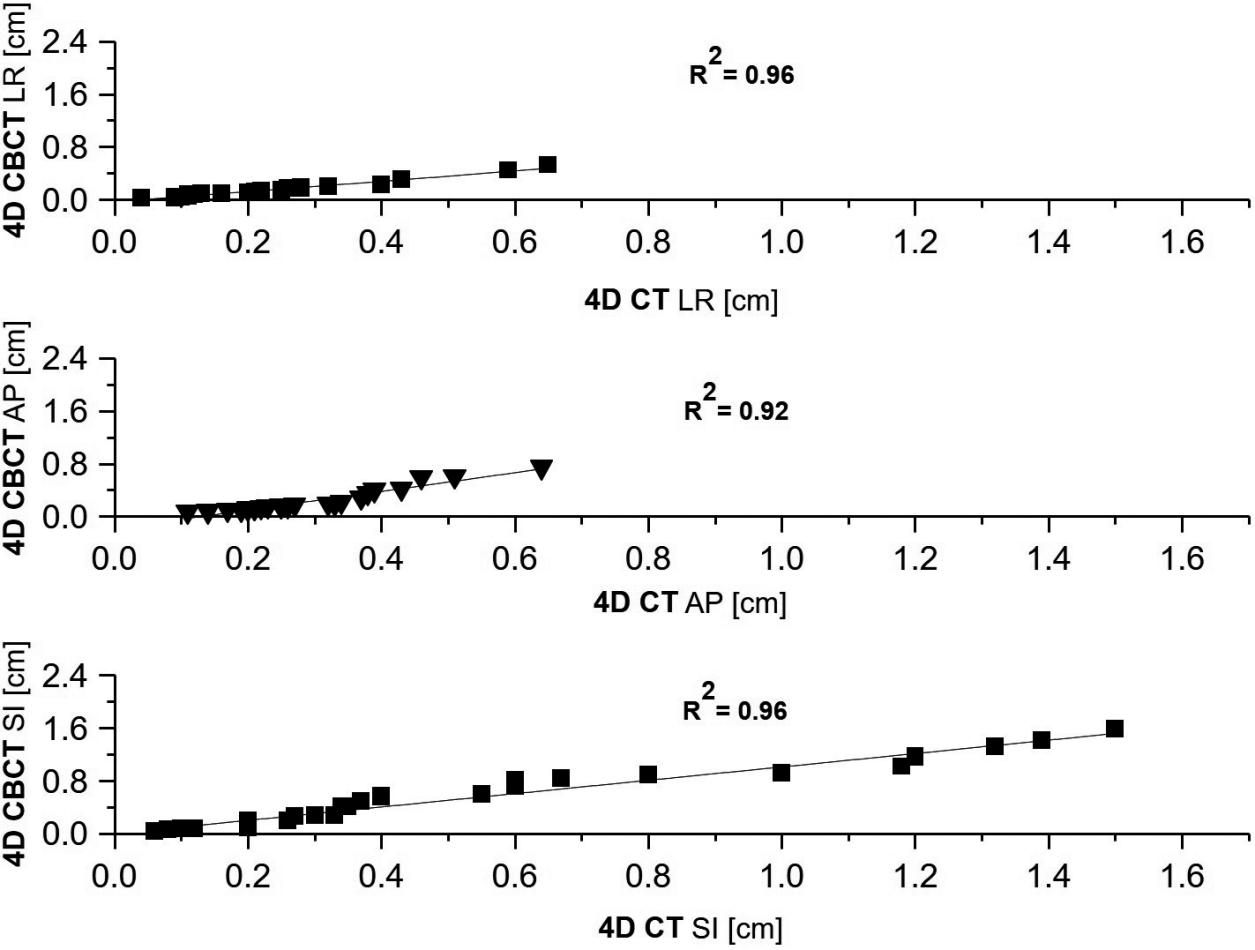
Correlation between 4D CT and 4D CBCT target motion displacement in all directions. The dashed‐dotted line corresponds to a unity fit.

Figure 1 shows the relation of the target motion between 4D CT and the first 4D CBCT in the three directions. The correlation coefficient was R^2^ = 0.96 in the LR and SI directions, and R^2^ = 0.92 in the AP direction.

### 3.2 Error analysis

**Fig. 2:**
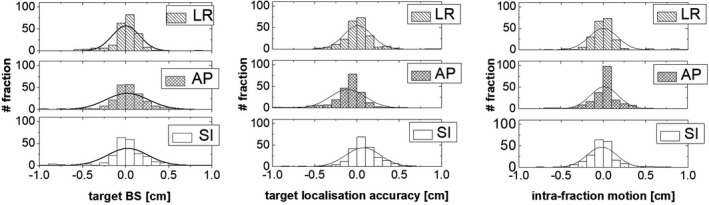
Histograms of the target baseline‐shift (left), target localization accuracy (centre), and intra‐fraction motion (right), in the three directions. The solid curves represent a gaussian fit of the data.

Figure 2 shows the histograms obtained from the analysis of the target baseline shift, the target localisation, and the intra‐fraction motion errors on the 229 fractions, in the three directions. The mean and standard deviation of the target baseline shift were 0.00 ± 0.16 cm, 0.02 ± 0.25 cm and 0.01 ± 0.22 cm for LR, AP, and SI directions, respectively. The mean and standard deviation of the target localization were −0.01 ± 0.16 cm, −0.11 ± 0.21 cm and 0.06 ± 0.21 cm for LR, AP and SI directions, respectively. The mean and standard deviation of the intra‐fraction were −0.00 ± 0.17 cm, −0.02 ± 0.18 cm, and 0.01 ± 0.15 cm for LR, AP and SI directions, respectively.

### 3.3 Margins computation

As expected,^26^, ^27^ the systematic errors are dominated by the delineation error (Table 2). All errors used for treatments (data from literature) were in general smaller than the ones calculated retrospectively (off‐line analysis) with the exceptions of the intra‐fraction in the LR and AP directions for the systematic error and AP direction for the random error (Table 2).

**Fig. 3:**
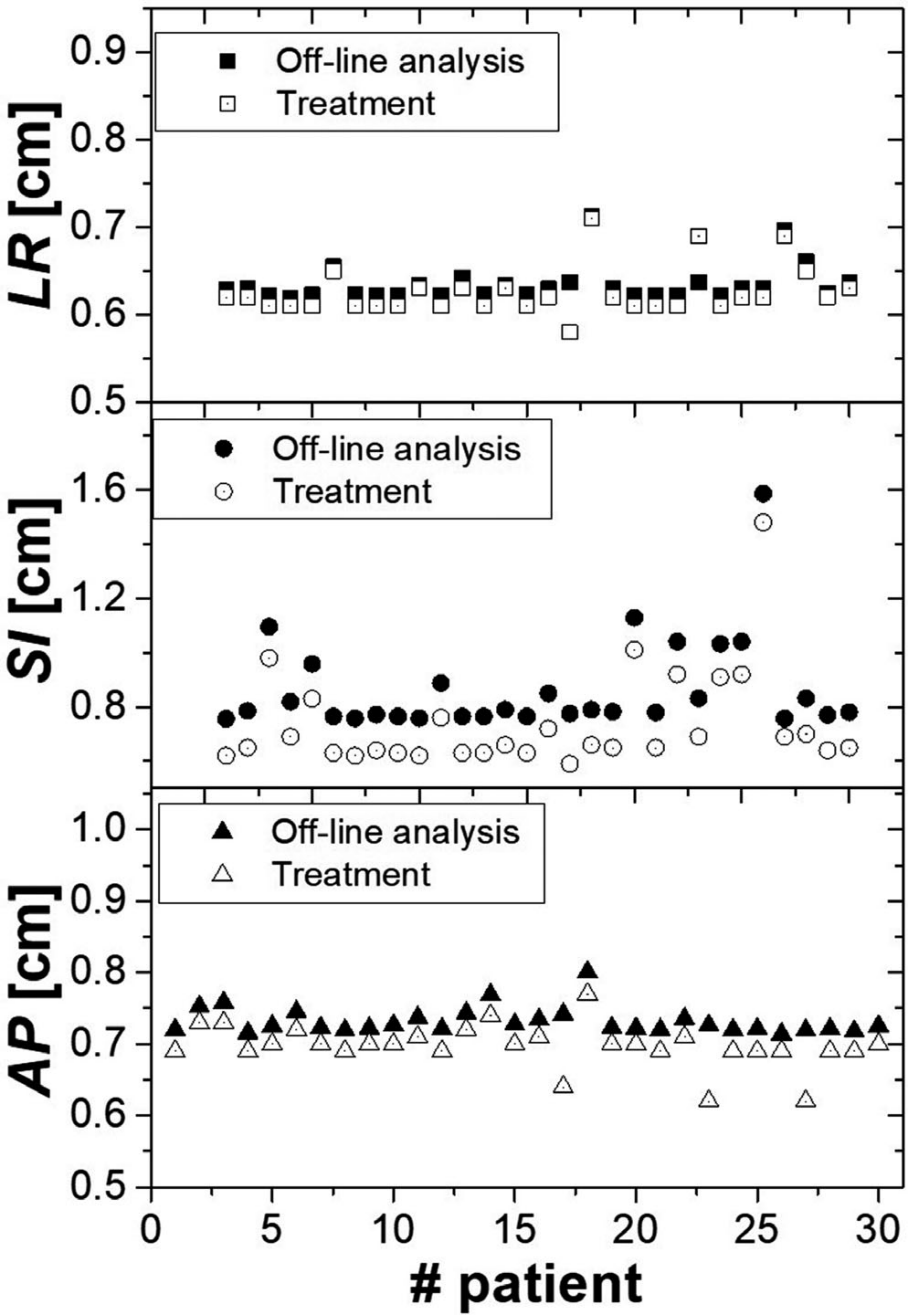
PTV margins calculated according to the off‐line analysis (4D CBCT data; filled marker) and from treatment (literature data; empty marker), in the three directions and for the 30 patients, respectively. Abbreviations: LR, Left‐Right; AP, Anterior ‐Posterior; SI, Superior‐Inferior.

Figure 3 shows the PTV margins based on published data and off‐line data from 30 patients treated at our center. The margins calculated with off‐line data were systematically larger than those coming from the literature.

### 3.4 Iterative analysis

**Fig. 4:**
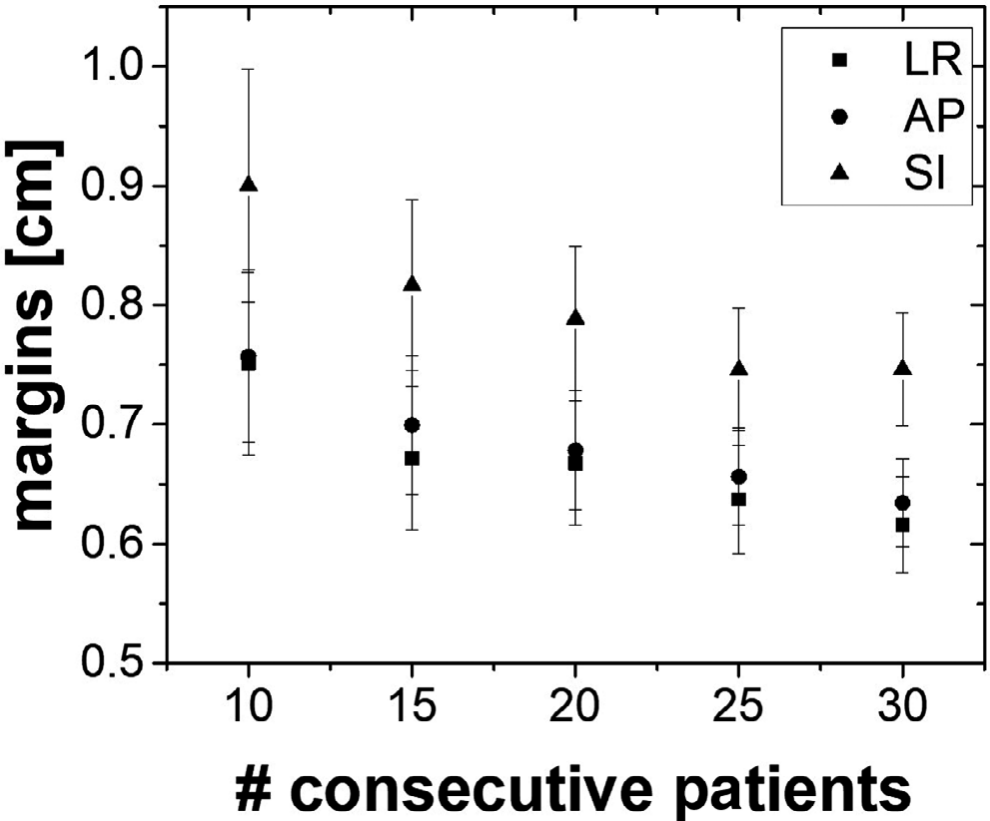
Margins calculated from the off‐line analysis (4D CBCT data) for a different number of patients. The error standard bars for each data are also shown in the graph. Abbreviations: LR, Left‐Right; AP, Anterior ‐Posterior; SI, Superior‐Inferior.

The margins calculated with the iterative analysis using different subsets of patient samples are reported in Figure 4, along with their corresponding standard error. An asymptotic margin reduction trend is observed as the number of patients involved in the calculation increases.

### 3.5 Bootstrap

**Fig. 5:**
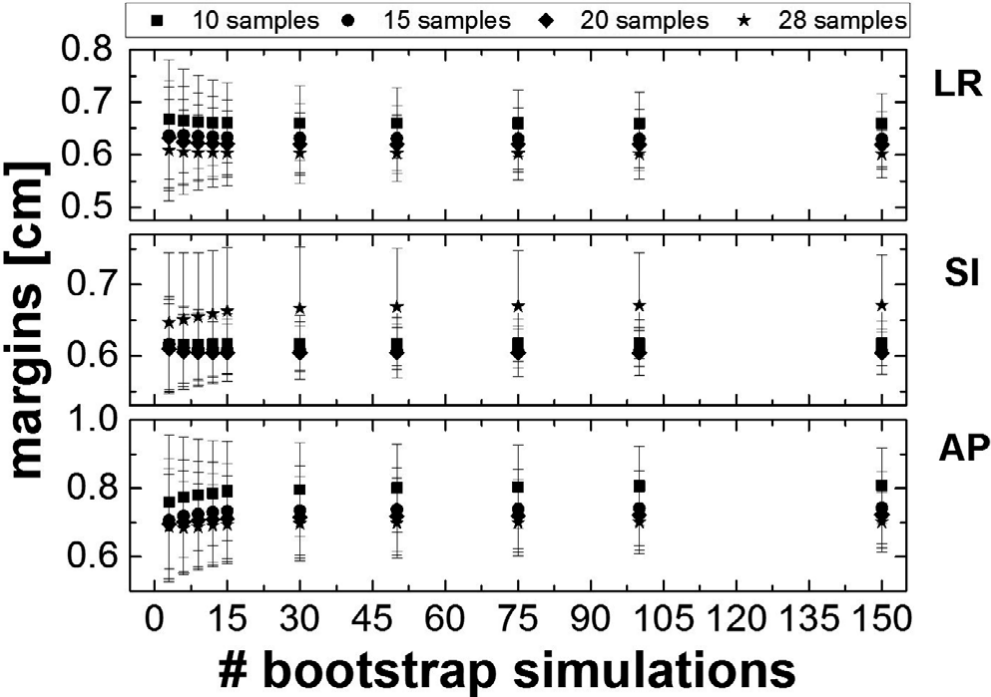
PTV margins calculated from the bootstrap analysis on the 4D CBCT data, in the three directions and for the 3 to 150 bootstrap simulations according to different initial patient sample sizes, 10, 15, 20 and 28, taken consecutively. The standard error bars for each data are also shown in the graph. Abbreviations: LR, Left‐Right; AP, Anterior ‐Posterior; SI, Superior‐Inferior.

Figure 5 shows the results of the margins calculated applying the bootstrap method for different simulations (number of included patients being 3, 6, 9, 12, 15, 30, 50, 75, 100 and 150), along with their standard errors. We did not observe any statistical difference on the calculated margins for cohort sizes including more than 15 patients.

## 4 DISCUSSION

The aim of our study was to provide centers, which are either implementing frameless lung SBRT or still using literature margins, a method to calculate personalized margins. The method proposed in this paper takes into account changes in the process due to modification of contentions, treatment technique, etc. In those cases, it is crucial to verify the applied margins to ensure continued safe SBRT delivery. We therefore retrospectively analyzed our patient cohort to assess the margin uncertainties induced by respiratory motion during frameless stereotactic radiotherapy, and then compared them with already published data. We also determined the minimum number of patients required to validate our treatment margins based initially on literature data with an iterative method and a bootstrap method. The bootstrap method performed better than the iterative method. The former having larger data than the latter was capable to provide better statistics and so to better converge.

At first, we assessed the target motion excursion for each patient by analysing 4D CT data. As expected, we found that the most important motion was in the superior‐inferior direction, with a maximum tumor motion of 2.2 cm, in agreement with other authors.^13^, ^14^ The tumors located in the lower lobe were the most impacted by the large motion amplitude, while the upper and median lobes were giving similar results. In this specifc case, the reliability of the frameless approach with respect to the breathing managing technique might be questionable. However, in our cohort we found only one patient where the motion amplitude was superior to 2 cm that would have justified the benefit of using breathing management technique. Moreover, we found a correlation between motion excursion detected in the 4D CT and in the 4D CBCT data, with a correlation coefficient of R^2^ = 0.96 in the LR and SI directions, and R^2^ = 0.92 in the AP direction. The observed differences may be due to the different techniques used for phase sorting, i.e belt for 4D CT and diaphragm motion for 4D CBCT. Nevertheless, the respiratory motion assessed from the 4D CT scan was mostly reproducible during the treatment delivery for our patient cohort (see Figure 1).^28^, ^29^, ^30^, ^31^ As also found in a recent paper of Liang, et al,^32^ if the baseline shift can be corrected by treatment couch, the target motion range is comparable to the one assessed with the 4DCT. Regarding the use of the 4D CBCT, it should be noted that for an ITV approach, the 3D CBCT alone may be sufficient since the blurred target volume on the 3D CBCT can be associated to an ITV; however for a Mid‐V approach, a 4D CBCT is highly recommended as confirmed by others.^33^, ^34^, ^35^ From the retrospective 4D CBCT data analysis, we observed that the target delineation was the largest error as was found by others.^26^, ^27^ It is well‐known,^10^ that this systematic error has a big impact on the margin definition. Unfortunately, this error was not assessed in this study as well as the sigma penumbra and we consider those two as limitations of the study. From the analysis of the target baseline shift, the target localization, and the target intra‐fraction motion we found, in all directions, mean values of maximum 0.2 mm and standard deviations of maximum 2.5 mm. This indicates the robustness of the Mid‐V strategy associated with the van Herk equation (see Figure 2). The larger systematic errors found for the AP target localization are probably caused by the patient relaxing between the first and second 4D CBCT. The values obtained for the systematic and random localization errors, i.e., discrepancies between planned and actual tumor position after corrections have been applied, were comparable to what was found by Sonke et al.^17^ However, the applicability of the van Herk equation in the context of SBRT might be still questioned. Within this context, Ecclestone et al^36^ have recently investigated the robustness of the van Herk equation to variation in tumor size, motion amplitude, tissue density and plan technique in lung radiotherapy and they ensured the safe clinical application of the van Herk margins as no CTV under dosage was observed. Another aspect to consider in the van Herk equation is the difference of the number of fractions for SBRT over standard radiotherapy treatment. For this reason as reported by Leong et al.^37^ and van Herk et al,^38^ in Equation 1 an additional systematic error was taken into account, being the quadratic sum of the random errors divided by the square root of the number of fractions. Moreover, Sonke et al.^17^ suggested that if the errors are quantified from a limited number of fractions, this effect will be implicitly taken into account when calculating margins. Therefore, the above considerations justify the application of the van Herk equation to the Mid‐V approach for safe lung SBRT. In particular, the Mid‐V approach minimizes some of those systematic errors, such as target localization and baseline shifts errors, hence reducing the margins size for a better sparing of the organs at risk. It should be noted that mid‐position, a refinement of the Mid‐V concept, would have even more improved the image quality by means of artifacts reduction as described by Wolthaus et al,^39^ thus providing a better representation of the tumor shape and volume. However, this approach is more complex and time consuming since it requires a reliable deformable image registration tool. Nevertheless, Peulen et al^40^ have reported an excellent local control of 98% using the Mid‐V based PTV margin approach combined with online image guided SBRT in early stage lung cancer.

Senthi et al^41^ have reported in their sytematic review low level of toxicity with SBRT, offering a safe and effective curative treatment for patients with central tumors who unfit for surgery. In Figure 3 was shown the comparison between the applied systematic and random errors obtained from the literature (treatment) and the systematic and random errors calculated retrospectively from the 4D CBCT (off‐line analysis). The comparison illustrated that the treatment margins were systematically larger in all directions with the largest difference of about 1 mm in the SI direction. Our results seem to indicate that margins calculation depends on the local parameters of the patient cohort, treatment technique and immobilization system as described by Lin et al^42^ and that the parameters used in the literature may not be used locally without caution. In other terms, data from the literature might be taken as a starting point, but the margins applied to patients should be re‐evaluated with local results obtained through acquired experience (i.e. patient treatments). Not performing that local evaluation could lead to overly large margins that might, in certain cases, be clinically unacceptable for lung stereotactic treatments. For this reason, to assess the robustness of our results and to determine the minimum number of patients required to validate custom margins, the latters were calculated using first an iterative method. As shown in Figure 4, the results did not converge for 30 patients; in particular, a monotone decreasing function with associated large errors bars was observed. As a consequence, the bootstrap method was chosen, providing more statistics. This method was recently implemented in radiation oncology to estimate toxicity, setup errors, organ motion, or in other applications.^43^, ^44^, but it was never applied to the context of margin validation. The analysis showed that, independently from the initial patient sample size, a sample of at least 15 patients was needed to observe a convergence (see Figure 5). This is in agreement with a similar study conducted by Matsumoto et al,^45^ Chaikh et al,^46^ where they also reported that the bootstrap method can be considered as a practical solution to simulate a larger population in case of a small sample size. In their paper, the authors recommended the use of the bootstrap for a sample size larger than 10 to provide good estimation in case of heterogeneous and not normally distributed data. The main difference between the iterative and bootstrap methods is that with the iterative method we can only extrapolate the data to obtain the correct margins, while it is possible with the bootstrap method to reach a convergence (plateau in Figure 5) with at least 10 patients,^28^ and so to define the correct margins in each direction.

A limitation of this study is that rotational errors were not taken into account in the margins computation, because our linear accelerator was not equipped with a 6 degrees of freedom (DOF) couch. However, in our study we did not observe rotation angles larger than 1°. Besides, it is important to notice that the lung SBRT tumors are generally small and spherical in shape; thereby the value of rotational shifts is further minimized, by placing the isocenter within the GTV. Indeed, as found by Ottoson et al,^47^ CTV to PTV margins were approximately the same, regardless if the CBCT matches were performed with 3 or 6 DOF. Finally a robotic couch wouldn’t lead to further margin reduction.

If we now consider the impact of the type of technique VMAT or IMRT to the margins, in the publication of Rana et al^48^ it has been shown that the major advantage of VMAT over IMRT is in the reduction of the number of monitor units leading to a faster treatment delivery times without compromising the quality of the treatment plans. Hence, faster delivery time is more patient‐friendly and minimizes intra‐fraction patient motion enabling the reduction of the treatment margins.^49^, ^50^ In line with this study, in our center all SBRT are delivered with VMAT technique.

## 5 CONCLUSIONS

The study presented here is a retrospective margins analyisis collected on a group of patients. We believe that this analysis could be beneficial to centers that want to apply personalized margins or simply adapt their margins.

The minimum number of patients needed to reach a convergence in the margin definition was evaluated using both iterative and bootstrap methods. The two methods provided similar results within the uncertainties. However, the iterative method was not converging because the number of patients of our cohort was not enough to reach conclusions on the margins. On the contrary, the bootstrap method showed that the required patient sample size to validate our treatment margins for frameless lung SBRT on a population scale was 15 patients.

To conclude, when implementing lung SBRT treatments, or when adapting the lung SBRT treatment protocol due to changes in local practice, an analysis based on the bootstrap method is suggested to verify the safe delivery of the treatment, without adding extra dose to the patient and increasing the treatment time.
